# Surgical Diseases of Infants and Children

**Published:** 1873-02

**Authors:** 


*Surgical Diseases of Infants and Children.* By M. P. Guersant. Translated from the French. By Richard J. Dunglison, M. D. Philadelphia : Henry C. Lea, 1873. Buffalo: T. Butler & Son.

This work is divided into seventy-three chapters and treats of most of the surgical diseases incident to infancy and childhood. It is not, however, the intention of the author to treat of the entire range of infantile surgery, but rather to place before the reader the results of observation upon cases which have come under his own treatment. Of the more rare surgical affections of children he either speaks but briefly, or from lack of experience in their treatment ignores them altogether.

The arrangement of the different subjects, is perhaps, open to objection, no order being observed in the distribution of the chapters. Thus, for instance, we have Chapter IV., treating of Fractures; Chapter V., of Tracheotomy in Croup; Chapter XX., of Cataract, and Chapter XXI. of Abdominal Hernia. Some of the rules laid down for operation and treatment are perhaps somewhat behind the times, but they will be found in the main to be excellent and supported by ample and careful observation.

The American translator, Dr. Dunglison, has performed his task with credit, and placed the profession under obligations for his excellent translation of M. Guersant's work.

